# Supplementary material for: Risk Assessment for Heroin Use and Craving Score Using Polygenic Risk Score
Source: J Pers Med. 2021 Apr 1;11(4):259. doi: 10.3390/jpm11040259 (PMC8066654; doi:10.3390/jpm11040259)
Supplement: Supplementary file 1 [file jpm-11-00259-s001.pdf]

Supplementary Table1. Associations from single SNP analysis in reward pathway genes

SNPs	Alleles	Gene	chromosome	Position	Gene Pathway	p_{crude}	p_{adj} #
rs2240158	T>C	GRIN3B	19	1005231	Cognitive function	0.02318	0.0353
rs2285906	G>A	GRIN3B	19	1008684	Cognitive function	0.5066	0.6901
rs2285907	C>G	GRIN3B	19	1008880	Cognitive function	0.4094	0.5653
rs7030238	C>A	GRIN3A	9	101570213	Cognitive function	0.17943	0.105
rs1983812	G>A	GRIN3A	9	101570761	Cognitive function	0.19707	0.0962
rs942142	C>A	GRIN3A	9	101670591	Cognitive function	0.1968	0.1238
rs10512285	A>G	GRIN3A	9	101670752	Cognitive function	0.28622	0.2212
rs3983721	C>T	GRIN3A	9	101733129	Cognitive function	0.00945	0.0109
rs17078853	G>T	GRM6	5	178981601	Cognitive function	0.46362	0.4904
rs2071247	A>G	GRM6	5	178983150	Cognitive function	0.2372	0.2303
rs17078877	G>A	GRM6	5	178983212	Cognitive function	0.67266	0.7128
rs11746675	C>T	GRM6	5	178986946	Cognitive function	0.50054	0.4352
rs2067011	C>T	GRM6	5	178988936	Cognitive function	0.96561	0.7135
rs211105	G>T	TPH1	11	18033757	Dopamine and serotonin pathway genes	0.09052	0.0826
rs623580	T>A	TPH1	11	18042430	Dopamine and serotonin pathway genes	0.55638	0.2889
rs910080	A>G	PDYN	20	1979580	Opioid system genes	0.27167	0.1299
rs2235751	G>A	PDYN	20	1989288	Opioid system genes	0.61716	0.9415
rs1997794	T>C	PDYN	20	1994212	Opioid system genes	0.36247	0.401
rs2129575	G>T	TPH2	12	71946293	Dopamine and serotonin pathway genes	0.03343	0.0474
rs1386493	C>T	TPH2	12	71961399	Dopamine and serotonin pathway genes	0.47575	0.4499
rs2171363	T>C	TPH2	12	71966484	Dopamine and serotonin pathway genes	0.3577	0.2229
rs7305115	A>G	TPH2	12	71979082	Dopamine and serotonin pathway genes	0.38303	0.2247

rs10506645	T>C	TPH2	12	71991720	Dopamine and serotonin pathway genes	0.83583	0.7553
rs4760820	C>G	TPH2	12	72003216	Dopamine and serotonin pathway genes	0.2774	0.3601
rs9325202	A>G	TPH2	12	72013697	Dopamine and serotonin pathway genes	0.45861	0.3986
rs1487275	G>T	TPH2	12	72016512	Dopamine and serotonin pathway genes	0.64424	0.5455
rs1487275	G>T	TPH2	12	72016512	Dopamine and serotonin pathway genes	0.64424	0.5455
rs2020917	T>C	COMT	22	19941361	Dopamine and serotonin pathway genes	0.36261	0.34
rs933271	C>T	COMT	22	19943884	Dopamine and serotonin pathway genes	0.28089	0.3739
rs174675	C>T	COMT	22	19946528	Dopamine and serotonin pathway genes	0.97392	0.8936
rs5993882	G>T	COMT	22	19950010	Dopamine and serotonin pathway genes	0.8531	0.6323
rs174699	C>T	COMT	22	19966935	Dopamine and serotonin pathway genes	0.10576	0.069
rs174699	C>T	COMT	22	19966935	Dopamine and serotonin pathway genes	0.10576	0.069
rs806368	C>T	CNR1	6	88140381	Opioid system genes	0.93632	0.9788
rs806380	A>G	CNR1	6	88154934	Opioid system genes	0.94548	0.8979
rs6473799	G>A	OPRK1	8	53240563	Opioid system genes	0.37833	0.3197
rs7271530	C>T	OPRL1	20	64096562	Opioid system genes	0.1213	0.1215
rs11528090	G>T	CYP2C1	10	94838682	Methadone-metabolizing enzymes	0.13323	0.1388

rs6010717	C>G	OPRL1	20	64083430	Opioid system genes	0.31926	0.3722
rs6583954	T>C	CYP2C1	10	94774506	Methadone-metabolizing enzymes	0.05439	0.033
rs16974799	C>T	CYP2B6	19	40998172	Methadone-metabolizing enzymes	0.4281	0.4704
rs3760657	A>G	CYP2B6	19	40989528	Methadone-metabolizing enzymes	0.64881	0.4632
rs2229205	T>C	OPRL1	20	64098078	Opioid system genes	0.95591	0.9669
rs4646425	C>T	CYP1A2	15	74750940	Methadone-metabolizing enzymes	0.60777	0.678
rs2236860	A>G	OPRD1	1	28814236	Opioid system genes	0.94117	0.952

[#] General linear model for association between Heroin using and craving score and genetic SNPs with adjustment for age, BMI and sex.
